# Mortality, morbidity and health in developed societies: a review of data sources

**DOI:** 10.1186/s41118-018-0027-9

**Published:** 2018-01-29

**Authors:** Guillaume Wunsch, Catherine Gourbin

**Affiliations:** Centre for Demographic Research, Catholic University of Louvain, Place Montesquieu 1/L2.08.03, B-1348 Louvain-la-Neuve, Belgium

**Keywords:** Mortality, Morbidity, Health, Data sources, Record linkage, Big Data

## Abstract

The purpose of this paper is to review the major sources of data on mortality, morbidity and health in Europe and in other developed regions in order to examine their potential for analysing mortality and morbidity levels and trends. The review is primarily focused on routinely collected information covering a whole country. No attempt is made to draw up an inventory of sources by country; the paper deals instead with the pros and cons of each source for mortality and morbidity studies in demography. While each source considered separately can already yield useful, though partial, results, record linkage among data sources can significantly improve the analysis. Record linkage can also lead to the detection of possible causal associations that could eventually be confirmed. More generally, Big Data can reveal changing mortality and morbidity trends and patterns that could lead to preventive measures being taken rather than more costly curative ones.

## Introduction

The purpose of this paper is to review the major sources of data on mortality, morbidity and health in Europe and in other developed regions in order to examine their potential for analysing mortality and morbidity levels and trends in developed societies and to inform healthcare and health policies. The value of statistical information for improving health policies is thoroughly discussed in Egidi and Buratta (2006). Our aim is to show the more relevant sources for evaluating health, morbidity and mortality at the individual level. Many references are given to the Nordic countries, as their data sources are among the best available, but the present paper also refers to a variety of innovative data sources and analyses in other European countries and abroad. As pointed out below, no attempt is made to cover the developed countries exhaustively. The review is based on papers published in demography, epidemiology, and public health and on information drawn from the websites of the organisations collecting the data. The paper is primarily focused on routinely collected information covering a whole country or a major part of a country, in contrast to surveys for specific populations such as physician surveys. No attempt is made to draw up an inventory of sources by country; this would indeed be a useful future research project. The paper deals instead with the pros and cons of each source for mortality and morbidity studies in demography. Data sources for evaluating healthcare performance and costs—such as in the EuroHOPE project—lie outside the scope of this paper, though these are of course crucial issues.

A variety of sources of data on morbidity, health and mortality are available in developed societies. While each source has its own advantages and problems, some problems are common to a large majority of sources, such as population selection and representativeness, non-response and inadequate reporting, privacy rights and related ethical issues, cost of large operations and cross-country comparability. Specific issues will be pointed out for each source. As stated in the conclusion, each source considered separately can already yield useful, though partial, results that can be used for research purposes and for informing health policies. Record linkage among data sources can significantly improve the analysis even further. Record linkage can also lead to the detection of possible causal associations that could eventually be confirmed. More generally, Big Data can reveal changing mortality and morbidity trends and patterns that could lead to preventive measures being taken rather than more costly curative ones.

The order of presentation is as follows: The different sources of data on mortality are analysed in the ‘Data sources: mortality’ section. The ‘Data sources: morbidity’ section reviews the major sources of data on morbidity. In the ‘Data sources: health status’ section, the data sources on health status are examined. The ‘Record linkage of multiple sources of data’ section discusses the use of record linkage of multiple sources of data for mortality and morbidity studies, while the ‘A Big Data approach’ section points out some issues of a Big Data approach. The concluding ‘Conclusions’ section discusses matters relating to the use of record linkage and Big Data.

## Data sources: mortality

This section deals with the vital registration of death and causes of death, including external causes of death. It also refers to postmortem examinations that can improve cause-of-death reporting.

### Vital registration: the death certificate and registration of causes of death

As Brolan et al. (2017) point out, it is continuity, completeness and relevance at local administrative levels that distinguish civil registration and vital statistics information from other population data sources. Statistics on causes of death, used to measure the relative contributions of various diseases on mortality, are based on the death certificate delivered by a medical doctor. For an overview of the data production process and the main types of possible analyses see, e.g. Rey (2016). In the cases where the patient dies at the hospital or the doctor certifying the death is the patient’s general practitioner (GP), the information on the cause of death is supposed to be reliable. However, and especially for the GPs, the question is raised about their training in terms of knowing how to fill in the medical certificate and their interest in doing so (McAllum et al. 2005). As an example of a special training programme addressed to students of medicine in their last year, family doctors and interns, with the objective of improving professional competence regarding the certification of causes of death according to the international regulations of the World Health Organisation (WHO), see Alonso-Sardón et al. (2015).

Most European countries have adopted the use of the medical certificate of causes of death proposed by the WHO. This certificate was proposed in 1977 (at the time of the International Classification of Diseases ICD-9) in order to ensure a better comparability of the statistics on this subject between countries. Causes of death should now be coded following the International Statistical Classification of Diseases and Related Health Problems (presently ICD-10), see Meslé (2006) for a comprehensive overview. The certificate is set out in two parts. The first part is designed to retrace the process which has led to death. The starting point is the immediate, or direct, cause of death (line 1). Then successive lines invite going back through the sequences that led to death, the last line of part 1 providing the underlying cause of death, and the lines between the first and the last delivering the intermediate cause(s). Part 2 of the death certificate is devoted to condition(s) which could have contributed to death but are not part of the main causal sequence leading to death. Finally, on the right-hand side of the certificate, a column allows stating the approximate time interval between the onset of each condition and death, in order to verify the coherence of the description of the process.

Statistics of causes of death are based on the *underlying cause of death*. WHO defines this underlying cause of death as ‘(a) the disease or injury which initiated the train of morbid events leading directly to death or (b) the circumstances of the accident or violence which produced the fatal injury’ (WHO 1992a, p. 1235). WHO has also proposed Injury Surveillance Guidelines (2004) for recording non-fatal events by injury surveillance systems; the latter will not be considered in this review. Using the WHO classification in force (currently, ICD-10—an ICD-11 is in its final phase and is planned for implementation in 2018), the reported conditions are translated into medical codes. WHO (1992b) has formulated selection and modification rules in order to improve the reliability of mortality statistics and to allow selecting a single cause of death, the underlying cause, from a reported sequence of conditions. All morbid conditions, diseases and injuries entered on the death certificate represent the *multiple causes of death* (see ‘Using data on multiple causes of death’ section below).

An increasing number of countries are adopting the coding software Iris; this software ensures a high international comparability of cause-specific mortality data. Iris is a result of the international collaboration of several national statistical institutes for the selection of the underlying cause of death on the basis of ICD-10. The user enters ICD-10 codes corresponding to the conditions reported on the death certificates. Iris then selects the underlying cause (IRIS Institute Website, http://www.dimdi.de). One can point out the pioneering role of the USA in implementing automatic coding, with the National Center for Health Statistics’ development in the late 1960s of the ACME computer system for standardising the production of mortality statistics.

The content of the death certificate, and its possible routine linkage with other data sources, allows the institutions in charge in each country to produce statistics on causes of death according to several socio-demographic characteristics (see ‘Record linkage of multiple sources of data’ section). These are relevant for identifying vulnerable populations and factors associated with important risks against survival, possibly leading to the design of public health policies.

As already stated, information published by statistical offices on causes of death usually relates to the underlying or initial cause of death. Though the data on underlying causes of death are not comparable over long periods of time due to changes in the WHO’s International Classification of Diseases (ICD) and to improvements in diagnostic practices, they do shed some light on time trends for the large ICD chapters of causes of death. There are presently 20 chapters of causes in ICD-10. The underlying cause of death can be used to analyse mortality differences between populations or over time. For example, racial differences in life expectancy in the USA can be ‘explained’ by the differences in the leading causes of death between the white and the black populations (Kochanek et al. 2013). Policies can then be set up to close the gap by addressing specific causes of death.

Several attempts have been made to bridge different ICDs by the reclassification of causes according to one of the ICD revisions. Probably, the best known of these attempts are those by France Meslé and Jacques Vallin, with various collaborators, at the INED in Paris (see, e.g. Meslé and Vallin 1996). The registration of causes of death is not without problems, however, and these can hamper comparisons over time and across countries, as when using the WHO Mortality Database on mortality by age, sex and cause of death. First, one can point out the successive revisions of the ICD and the problem of bridging between two ICDs. For example, if one compares ICD-10 to ICD-9, ICD-10 not only increases the classification details (and shifts from a numerical to an alphanumerical classification system) but also includes ‘changes in the coding rules by which a single cause of death is selected from among the multiple causes reported by physicians as causing or contributing to the death’ (http://cancerprofiles.ca). Secondly, changes in the reports of causes of death may also be due to the use of new diagnostic techniques allowing a more efficient detection of diseases, to changes in the concept of diseases, and the appearance of new diseases such as HIV. Thirdly, training of MDs for the certification of causes of death can vary greatly among countries. In addition to the diagnostic accuracy issue, MDs only report diseases or conditions that they judge relevant for causing the death. According to WHO, the rate of ill-defined causes per 100,000 people, at ages 0 to 64, varied from 0.44 (Malta) to 22.51 (Portugal) for the EU population (European Health Information Gateway, 2012 data). In addition, age misreporting can sometimes be a problem for some population groups, such as immigrants. Finally, even among developed countries, the coverage of the cause-of-death statistics can vary across countries and over time. For example, according to recent estimates, the coverage was estimated for Cyprus at 78% in 1997 and at 86% in 2011, while it was 100% at both dates in Sweden (World Health Organization, Global Health Observatory Data Repository/World Health Statistics). And one should also point out the difficulty, as with all classifications, in reaching univocal solutions in the ICD.

### Using data on multiple causes of death

As seen in the previous section, contributory causes of death are also recorded on the death certificate, in addition to the underlying cause. One can, for example, study hypertension-related deaths by taking into account any mention of hypertension on the death certificate, using multiple cause-of-death data. Though useful for determining associations among causes of death, causal sequences and patterns of diseases (see for instance Redelings et al. 2007), contributory causes of death are however rarely considered in the tabulation of mortality statistics, though death is often the result of a combination of causes, especially at older ages. In their book *Recent Trends in Mortality Analysis*, Manton and Stallard (1984) were among the first to thoroughly examine the use of multiple-cause mortality data in the context of medical demography. In particular, they examined *pattern-of-failure* representations of mortality, i.e. combinations or joint occurrences of causes of death found on the death certificates, and showed the advantages of this approach compared to the sole use of the underlying cause of death.

A review of studies on the use of multiple causes of death is presented in Désesquelles et al. (2012). The authors also took up, to give an example among others, the issue of analysing multiple causes of death (MCOD), with an application to cancer-related mortality in France and Italy. After studying the quality of the data on multiple causes of death, the authors present various indicators for analysing MCOD data. Using French and Italian data, they examine the most frequent associations of causes of death with cancer, the latter being reported either as the underlying cause of death or as a contributing cause. Five patterns of associations are distinguished in the present case. The authors conclude by stressing the interesting fact that the multiple-causes-of-death approach ‘can help to identify associations which, though not currently validated by medical knowledge, should be taken seriously and investigated further’ (p. 488). Désesquelles et al. (2014) also used the multiple-cause-of-death approach to compare mortality from Alzheimer’s disease, Parkinson’s disease and dementias in France and Italy, pointing out inter alia possible dissimilarities of reporting practices. Though the multiple-cause-of-death approach can be recommended, the number of contributory causes reported on the death certificate varies from country to country, hampering to some extent international comparisons. Differences among countries with respect to certification and coding systems can be a problem.

### External causes of death

While in the previous section the focus was on the disease as a cause of death, the monitoring of external causes of death is also an essential requirement for public health and policy purposes aimed at injury prevention. For example, falls and suicides are an important cause of death in the elderly; the death toll from traffic accidents is high among the young. As mentioned above, according to WHO, the underlying cause of death consists in the present case of ‘the circumstances of the accident or violence which produced the fatal injury’ (WHO 1992a, p. 1235). The main external causes dealt with in ICD-10 (chapter XX) are accidents (transport accidents and other external causes of accidental injury such as falls), intentional self-harm, assault, operations of war and complications stemming from medical and surgical care. In addition, one should state the place of occurrence of the external cause where relevant and the activity of the person at the time the event occurred.

External cause-of-death registration is not without its problems, however. For example, an international study from 2000 onwards has shown that out of 83 countries having cause-of-death registration, only 20 countries had high-quality death registration data that could be used for estimating injury mortality, because elsewhere, injury deaths were frequently classified using imprecise, partially specified categories (Bhalla et al. 2010). Furthermore, it is well known that some types of external causes are difficult to evaluate. For example, suicides are often underreported, especially in countries where suicides are not morally or socially accepted (Tøllefsen et al. 2012). It is also difficult in many cases to determine the intent of the death: intentional self-harm, or homicide or accident…? Such is the case, for example, of single-vehicle accidents and drownings. To take another example, a death from a vehicle accident might occur not on the scene but some hours or days after the accident. What lapse of time after the accident should one choose after which the death will no longer be determined as resulting from that accident? This criterion varies between countries, from ‘died on the scene’ to ‘unlimited’, biasing international comparisons. WHO uses a 30-day time limit in its publications, adjusting, when possible, the data provided by national sources. Consider another case: that of pedestrian fatalities. There are several definitions of who is a pedestrian, and how pedestrians are defined has an effect on the number of deaths that are counted as pedestrian deaths (see Noland et al. 2017).

In order to improve data collection, some countries have set up special registration systems for covering violent deaths or have linked data from various sources. For example, in the USA, the National Violent Death Reporting System links data on violent deaths (e.g. suicide, homicide, legal intervention) from death certificates, coroner/medical examiner reports and law enforcement reports for the 17 participating states.^1^ In Brazil, data from the Hospital Information System, Mortality Information System and Police Road Traffic Database of five state capitals have been linked to improve information on the underlying cause of death, cause of injury and severity of injury in victims (Mandacaru et al. 2017). Record linkage is discussed in more detail in the ‘Record linkage of multiple sources of data’ section.

### Autopsies/postmortem examinations

Postmortem examinations, also called autopsies, can improve the quality of cause of death registration, e.g. in the case of sudden death. Ylijoki-Sørensen et al. (2014) have investigated ill-defined and unknown causes of death in Denmark and Finland. A forensic autopsy was performed in 88.3% of Finnish R00–R99-coded deaths, whereas only 3.5% of Danish R00–R99-coded deaths were investigated with the forensic or medical autopsy. Their study shows that if all deaths in all age groups with unclear cause of death were systematically investigated with a forensic autopsy, only 2–3 per 1000 deaths per year would be coded as having an ill-defined and unknown cause of death in national mortality statistics. At the same time, the risk of unnatural deaths being overlooked significantly decreases. To achieve this, in Europe, it would require that the existing legislation on cause-of-death investigation be changed to ensure that all deaths of unknown cause be investigated with a forensic autopsy.

Actually, according to the information available, it seems that postmortem examinations are routinely performed in only a few countries, e.g. Iceland. In most countries, they are executed solely in case of unexpected or (especially) suspicious death. Performance of autopsy is notified on death certificates in various countries. In Belgium, the requirement for an autopsy was notified on 1.5% of death certificates in 2010. In France, in 2011, the percentage was similar. However, notification is absent in 26% of cases in Belgium and 10% in France. When performed, autopsies may lead to changing the cause of death, and the registration system should be adapted to take this new information into account. To give an example of the procedure, in France (and also in Belgium), the autopsy is usually requested by the MD filling in the death certificate. In France, this request must be accepted by the coroner. Following postmortem results, the coroner fills in a second death certificate, cancelling the first.

Even if autopsies lead to improving cause-of-death registration, they raise several problems. Legal aspects have been pointed out above. Another issue is psychosocial—when the consent of the family is required, MDs can hesitate to seek it of the family grieving the death of a close relation, the representation of the autopsy act remaining very negative in most countries (Becart-Robert 2015). Another problem is the cost of an autopsy for the health system.

To conclude this section, vital registration data have been used for decades for constructing life tables all causes and by cause. Taking multiple causes of death into account could improve the picture by pointing out possible associations among causes of death.

## Data sources: morbidity

This section discusses the main data sources on morbidity. These sources include the surveillance of infectious diseases; sentinel networks; specific disease registers such as cardiovascular diseases and cancer registers, hospital statistics and general practice records, and insurance statistics.

### Surveillance of infectious diseases

Though mortality from infectious diseases is low in developed countries compared to that from chronic diseases, this does not imply that infections have been overcome. Children, pregnant women, older individuals and people with pre-existing diseases are particularly vulnerable to infectious diseases. Furthermore, infections can lead to sepsis and septic shock, which can be lethal in the older population especially. New forms of infectious diseases have appeared in the world, such as AIDS, SARS and Ebola. The incidence of some infectious diseases that were deemed conquered—such as tuberculosis—has increased significantly, and some diseases—such as influenza—are still highly lethal in some years, especially for the young and the old. Infectious agents, such as *Helicobacter pylori* and *human papillomavirus*, can also be a cause of various cancers. In addition, some pathogenic bacteria have become resistant to the drugs that are used to kill them.^2^

A list of notifiable diseases has been established by WHO, and countries have adopted for healthcare providers (treating physicians, diagnostic laboratories, hospitals, street-based mobile units) a compulsory declaration of various infectious diseases (single cases or outbreaks) that may constitute public health emergencies.^3^ As an example of current developments in this field, Denmark has recently set up a nationwide Danish Microbiology Database in order to enable real-time surveillance of communicable diseases and microorganisms (Voldstedlund, Haarh, Mølbak and MiBa Board of Representatives 2014).

To take into account the growth in international travel and trade, to which one can add changes in environmental conditions—including climate change—impacting on vector-borne diseases, in 2005, WHO revised its International Health Regulations (IHR), which entered into force in 2007 (WHO 2005). In addition to the notifiable diseases, these call for any event of potential international public health concern to be declared, including those of unknown causes or sources and those involving events or diseases other than those listed, if the public health impact is serious. The IHR are thus no longer restricted to a specific set of infectious diseases. According to WHO, with follow-up reporting in 2014, only 64 nations (33%) reported that they had fully implemented the IHR. The other 67% of the nations either requested another 2-year extension (81) or reported nothing at all (48) (Katz and Dowell 2015).

In the EU, a strategy for infectious disease surveillance was developed in 2005. This led to the creation, in Stockholm, of the European Centre for Disease Prevention and Control (ECDC).^4^ The Centre’s mission is to identify, assess and communicate as rapidly as possible among EU countries current and emerging threats to human health posed by infectious diseases. It gathers surveillance data from the EU Member States on 52 communicable diseases and works in partnership with national health protection bodies across the European Union to strengthen and develop continent-wide disease surveillance and early warning systems. Thanks to this, one can detect for example clusters of incident cases (such as a food-borne infection due to *Listeria*) and locate their source,^5^ or observe multiple cases of a particular communicable disease in different countries and trace them back to common international travel on the part of the individuals concerned. New technologies for identifying pathogens^6^ reduce the time needed for detection.

Accuracy, the timely reporting of cases, and the prioritising of threats are critical for communicable disease control. Early detection can mean the difference between an outbreak and a pandemic. As the more severe cases are hospitalised, reported cases can be checked against hospital discharge data. Boehmer et al. (2011) have shown for example that in the USA (Colorado), sensitivity and timeliness differed greatly among the notifiable diseases examined. For example, hepatitis A was poorly reported, while reporting was high for salmonellosis. The authors recommend the use of both medical records and hospital discharges for evaluating the quality of reporting. International comparisons may be hampered in Europe by the fact that reporting diseases not on the IHR list depends upon different national practices and laws. Finally, it can happen that countries fail to inform others of the occurrence of a disease.

We end this section by pointing out that environmental health surveillance systems have been set up to monitor environmental contamination, such as concentrations of contaminants in water and pollutants in ambient air, and more rarely to monitor contaminants in individuals. For example, the Flemish Environment and Health Survey on a representative sample of individuals collects data on biomarkers of exposure and effect, exposure-effect associations, time trends and geographical differences. The European Union launched in 2016 its European Human Biomonitoring Initiative for this purpose.

### Sentinel networks

According to the WHO,^7^ a sentinel surveillance system is used when high-quality data are needed about a particular disease that cannot be obtained through a passive system. A sentinel system involves a limited network of reporting sites, such as large hospitals or laboratories. For example, in Belgium, the surveillance of sexually transmitted diseases is carried out, i.a. through a network of voluntary-based sentinel microbiological laboratories evenly covering the whole country.

A particular case of surveillance systems is that of GP sentinel networks, composed of a sample of GPs on a voluntary basis. These networks allow a picture to be drawn of diseases widespread in the general population, usually not leading to hospitalisation, and estimating trends in, for example, the prevalence of diabetes. Diseases chosen for registration can vary over time. Taking the example of influenza, the European Influenza Surveillance Network is based on nationally organised networks of GPs covering at least 1 to 5% of the population in their countries, presently 31 EU/EEA Member States. These physicians report their weekly number of patients to their national focal point for influenza surveillance. The latter reports the data at the national level to the European Centre for Disease Prevention and Control and to the WHO Regional Office for Europe.

Several problems should be pointed out. Firstly, the actual reference population is unknown. When based on voluntary participation, the sample of participating units can be biased. For example, GPs participating voluntarily in a sentinel network may have practices and therapeutic schemes that are different from those of others; for example, they may be paying more attention to diagnostic precision. Furthermore, for some diseases such as migraines and depression, diagnostic criteria may be unreliable and vary greatly among GPs. Thus, the list of diseases chosen to be collected usually gives preference to pathologies which can be confirmed by biological, histological or radiological examinations.

### Specific disease registers

Following Rankin and Best (2014, p. 337), ‘a disease register is a documentation of all cases of a certain disease or health condition, which occur within a defined population. Registers are held by registries, which are the systems in place for the continuous registration of cases’. For example, the Finnish cancer register is maintained by the Cancer Society of Finland in Helsinki. Disease registers are either hospital- or population-based, an advantage of the latter over the former being the availability of denominator data for the base population. Two main examples of disease registers are presented in this section, on cardiovascular diseases and on cancer, as they represent the major causes of death in developed countries. Many other registers have been set up, e.g. on congenital abnormalities (chromosomal or not), such as the EUROCAT project in Europe. As a country example, there are over 200 registers in existence in England (Rankin & Best, op. cit.). This diversity of registers will not be considered here.

#### Cardiovascular disease registers

Registers of cardiovascular diseases (CVD) have been in operation since the early 1970s in various places, such as North Karelia and Turku (Finland) or Kaunas (Lithuania). The most important source of information on cardiovascular diseases has been the MONICA Project set up under the auspices of WHO. The MONICA Project was started in the early 1980s in various centres to monitor trends in cardiovascular diseases and to relate these trends to risk factor changes in the population over a 10-year period. A total of 32 MONICA Collaborating Centres were set up in 21 countries. Many of these registers are still in operation today. Other projects have started more recently. In particular, in Europe, the EURObservational Research Programme (EORP) was launched in 2010 under the auspices of the European Society of Cardiology. This programme presently covers 20 different registries, see the EORP website for further details (http://www.eorp.org).

As stated in the *MONICA Manual*, available online,^8^ four basic sources of information were to be used in the core MONICA study over a period of 10 years: (1) routinely available administrative data on the study population, from local government and local medical sources; (2) investigation of medically recognised cardiovascular events, fatal and non-fatal, using medical and medico-legal sources and validating the original diagnoses using MONICA criteria; (3) continuous or intermittent monitoring of the acute care of coronary and stroke events and (4) population surveys to monitor levels of risk factors and health-related behaviour. MONICA Centres were responsible for undertaking the registration of all coronary events within defined age groups (25–64 years of age, later shifted to 35–64) in both genders over a period of 10 years. Population risk-factor surveys had to be conducted at least at the beginning and at the end of this period, and optionally in the middle. Coronary care also had to be monitored, at least at the beginning and at the end of the period.

Several issues can be raised (Tunstall-Pedoe et al. 1994; Gourbin 1997; Tunstall-Pedoe 2003). A major difficulty of long-term projects such as the registration of diseases is to sustain the initiative over the years. According to Tunstall-Pedoe (2003, p. 127), in the case of MONICA, some centres failed to meet the deadlines for data while others discovered major problems with their data which have not been resolved, failed to obtain continuous funding for their local activities or simply lost contact, failing to reply to repeated communications.

As CVD registers often cover not a whole country but only a part of one, events can occur outside the population of reference. Access to hospital discharges outside the area of reference is therefore required. In addition, the same event can be declared by multiple sources, e.g. a hospital as well as a GP. It is thus necessary to link all events to the same individual by a unique identifier such as a personal identification number. Of course, one also has to have access to the cause of death declared on the death certificates, irrespective of possible privacy issues. Then there is the problem of diagnosis; diagnoses on hospital discharges or on death certificates do not necessarily correspond to the strict MONICA criteria for definite myocardial infarction and must be checked if possible. The MONICA Project showed that a large proportion of deaths had no relevant clinical or autopsy information.

Notwithstanding these issues, CVD registers remain an invaluable source of data for monitoring levels and trends in incidence and case fatality; reporting should therefore be mandatory. Recent methods of diagnosis both for coronary events and stroke now lead to much more trustworthy results than a few decades ago. Moreover, some registers—such as the Danish Heart Register and the Belgian Luxembourg-Province Register (BELLUX)—are now recording invasive coronary diagnostic and therapeutic interventions, in addition to myocardial infarcts (Abildstrøm and Madsen 2011; Jeanjean et al. 2012). It is relevant indeed to examine both care and outcome. Acute myocardial infarctions do not represent all the cases of coronary heart disease. It is therefore important to determine the angiographic appearance of coronary vessels. Registering both infarcts and interventions leads to a better estimation of the degree of atheromatosis and coronary heart disease in the population.

Due to the increase in life expectancy at older ages, the 64-year upper limit initially recommended by MONICA for registering myocardial infarctions is presently too low, and various registers have extended the age limit to 74 or more. Opting for these higher ages can be recommended, possibly pushing the upper age limit to 84, as BELLUX has done. However, above 85, it becomes difficult to disentangle the multiple pathologies often present in very old individuals.

#### Cancer registers

This section deals with population-based cancer registers, excluding among others hospital cancer registers that are mainly used for administrative purposes, and for which the catchment population is unknown. The purpose of a cancer register is to record new cases of cancer, i.e. incidence cases of cancer occurring in a defined population, in order to produce statistics on the occurrence of cancer (for example, see Arnold et al. 2015). A register can also eventually give information on the prevalence of cancer and on the survival of cancer patients if deaths from cancer and the possible emigration of cancer patients are known. Cancer registers can be either general, registering all tumours, or specialised, such as paediatric registers or registers restricted to one organ or tract. They can be managed inter alia by cancer societies, governmental agencies or public health institutes. Cancer registers can cover a whole country, as in many EU countries, or be geographically decentralised and coordinated at the national level, as in the USA.

In Europe, for comparison purposes, a European Cancer Observatory (ECO, http://eco.iarc.fr) has recently been set up, combining all the information currently available in Europe on cancer incidence, mortality, survival and prevalence. ECO is a project developed at the International Agency for Research on Cancer (IARC) in partnership with the European Network of Cancer Registries (ENCR). As of 2010, there were more than 200 cancer registers operating in Europe^9^; coverage, methods of data collection and data availability can however differ substantially among them (for an overview, see Siesling et al. 2015). In the specific case of childhood cancer, present registers cover 83% of the childhood population in the European Union and could increase to around 98% if the recently established registers start producing results and others improve their quality and dissemination plans (Steliarova-Foucher et al. 2015).

The main sources of information on cancer incidence are usually hospitals or cancer treatment centres, but many other sources may be involved, such as private clinics, GPs, practicing specialists, laboratories, screening programmes, death certificates, pharmaceutical prescription records and health insurance systems. Some cancer cases might only be detected on the death certificate. According to the International Agency for Research on Cancer, many such cases would be an indicator of poor reporting. Recourse to multiple sources of data implies that one has to handle possible multiple notifications of the same cancer case and that one must be able to link these notifications to the same individual, for example by way of a personal identification number (PIN). Multiple notifications can then be used to check the completeness of the registration. In several European countries, PINs are now attributed at birth to every citizen of the country. Record linkage among different data sources is covered in greater detail in the ‘Record linkage of multiple sources of data’ section. Nordic countries benefit on the whole from excellent cancer registers, due among other factors to favourable legislation.

One can use multiple sources for checking the completeness of reporting. In Finland, which has one of the best cancer registers, a recent study concluded that ‘the completeness for all solid tumours was estimated at 96%, and for non-solid tumours at 86%. Potential underreporting was most prominent for tumours which are not typically histologically verified’ (Leinonen et al. 2017). However, if cancers are well covered in good registers, the same cannot be said for benign tumours, such as benign pituitary tumours (predominantly microadenomas), the diagnosis of which is not necessarily coded in routinely collected data (Morling et al. 2016).

Some remarks can be raised on the basis of the registers operating in the Nordic countries, all of a very high quality. As the diagnosis of cancer relies on the WHO classifications of diseases (ICD), which are regularly updated, it is difficult to examine time trends in cancer incidence by site covering different revisions of the ICD. Diagnostic means have also evolved over time. Furthermore, as discussed above, completeness of registration remains a problem if one cannot cross-check the cancer register with other sources using record linkage. Notification must be legally mandatory, and reminders should be sent to sources which do not fully comply. The diagnostic information should be based on a histological examination by a pathologist. In Denmark, the proportion of morphologically verified tumours reaches 89%. Although already very high by international standards, this proportion could nevertheless still be increased (Gjerstorff 2011).

### Hospital statistics and general practice records

All hospitals collect information on their patients and on the medical, diagnostic and treatment services provided. An increasing number of hospital departments are adopting electronic health records (EHR) for their patients.^10^ Electronic health records, of course, require standardised structured patient data. Hospital statistics can be gathered into a national register. For instance, the Danish national patient register covers all somatic and psychiatric in- and outpatients. In Belgium, the Minimum Hospital Summary contains data both on each patient and on the hospital, for all hospitals except psychiatric ones. The latter collect their data via a Minimum Psychiatric Summary for each patient. The data collection is mandatory by law, and the individual data are anonymised. The purpose of these statistics is mainly to monitor hospital costs. In France, hospital data are collected by a survey aimed at all public and private hospitals and healthcare institutions. In Germany, data are collected by Länder, and delays may occur in providing federal results (Van de Sande et al. 2006).

For the purpose of morbidity/mortality studies, hospital data contain essential information. Hospital data serve as inputs for various disease registers, such as CVD, cancer and congenital malformation registers. Emergency department data can be used, for example, to study medically attended non-fatal injury episodes. Based on record linkage, these data can be complemented, as in the USA, by self- or proxy-reported information coming from the National Health Interview Survey, which includes calls to medical care providers, treatments at the scene, visits to clinics, emergency department visits and hospitalisations. Hospital electronic health records have been used in the UK, for example, to study why weekend hospital admission is associated with increased mortality, showing that this weekend effect arises from patient-level differences at admission rather than reduced hospital staffing or services (Walker et al. 2017).

Hospital records raise several issues. Only hospitalised patients are considered, and outpatients are not always included. Some hospitals may not participate in the system, such as private clinics, psychiatric hospitals or nursing homes. Diagnostic means can vary among hospitals. Without matching with a PIN, it is impossible to distinguish new patients from recurrent ones or to link these data with other sources. The reference population can be taken as the national population only if the system is exhaustive. Ethical issues concerning privacy and access, and matters concerning the rights, responsibilities and control of the system by physicians and patients, have to be solved (Garrety et al. 2014).

Concerning general practice records, the trend is towards recording patient information in an electronic format. This does not mean that the data are centralised at the national level. However, in Denmark for example, GPs provide daily information concerning patient visits to the National Board of Health. In England, the General Practice Extraction Service (GPES) collects information from the four general practice clinical systems. In some cases, such as in the Netherlands, data can be provided by the GP sentinel network (see ‘Sentinel networks’ section).

Some countries, such as England, have attempted to go further than a national register dealing solely with hospital statistics by setting up a national electronic health record system taking into account physicians’ electronic health records of their patients. Such a system includes not only hospital records but also electronic medical records held by GPs and specialists. In England, the linkage between general practice records and hospital admission data has led, among others, to improving incidence estimates of common conditions such as pneumonia (Millett et al. 2016). An electronic medical record system can enhance the share of patient information among healthcare providers. It can also improve the integration of social determinants of health into healthcare delivery systems (Gottlieb et al. 2015). One could possibly also include, if recorded, patient-reported outcomes, reflecting patients’ perspectives on their health (Bartlett and Ahmed 2017).

### Social security data and health/dependency insurance statistics

Though social security data are mainly concerned with costs and expenditures, they can sometimes be used for demographic purposes. For example, Lauderdale and Kestenbaum (2002) have used data from the US Social Security Administration for estimating age- and sex-specific death probabilities for the elderly of six Asian American subgroups, avoiding numerator/denominator bias when these are provided by different sources. In particular, the issue of determining age for the foreign-born and for immigrants was dealt with.

Public or private health insurance systems can sometimes provide useful information associated with morbidity. To give just one example relating to Belgium, the Intermutualist Agency was founded in 2002 to collect information on medical visits (to GPs, specialists, nurses, etc.) and information on prescribed medication and health interventions (see De Grande et al. 2014). All persons insured by the mandatory Belgian health insurance programme are covered.

Studies based on health insurance data can relate to the onset of handicaps, hospital admissions, preventive dental care, etc. Administrative and billing data can be used to study, for example, social inequalities in health or differences in health status between regions. For example, in the USA, Medicare data can be used for comparative effectiveness research on treatments, benefit designs and delivery systems for Medicare beneficiaries (as determined by the Social Security Administration), i.e. around 50 million people nationwide (see for instance Mohr 2012). These data permit, for example, national assessments of imaging utilisation and spending for this subpopulation. A French study has used healthcare consumption to compare the health status of beneficiaries of the French national health insurance general mandatory scheme between individuals living in French overseas territories and those living in metropolitan France. The data were extracted from the French national health insurance database (SNIIRAM) for 2012 (Filipovic-Pierucci et al. 2016). SNIIRAM (*Système national d’information interrégimes de l’Assurance maladie*) is a French medical-administrative database containing all reimbursements provided by the national health insurance scheme for each individual contributor, over their whole life. France is also setting up, from an open-access perspective, a national system of health data (*Système national des données de santé*), of which SNIIRAM will be the cornerstone, that will also contain the anonymised health data (causes of death, social and medical data and complementary health insurance) collected by various public institutions.

A special case in terms of public^11^ health insurance is dependency insurance. Very few countries have set up such a public system at the national level. In the EU, the pioneers in this field have been Austria (since 1993), Germany (since 1995) and Luxembourg (since 1999). Most ages are covered in these systems. In each case, an assessment of individual needs for long-term care is first conducted, either by a multidisciplinary evaluation unit or a physician, and provision of care is planned either in cash or in services on the basis of limitations in the activities of daily living. For the population covered by the programme, dependency insurance systems can provide (as in Luxembourg) the causes of dependency according to age, sex and residence, among others. They can also show the costs of care (including informal care) and the type of services dispensed.

Using health insurance data can nevertheless be problematic. Firstly, individuals not covered by health insurance are of course not included. Secondly, the sources primarily provide financial data rather than health and morbidity data. Thirdly, if the insurance schemes are privately run, it can often be difficult to obtain the required data. Finally, socio-economic individual data are frequently not included in the sources and the data have to be linked to other types of sources for this purpose, by way of a personal identification number (see ‘Record linkage of multiple sources of data’ section).

To conclude this section, many sources of individual data on morbidity are now available in developed countries. Taking advantage of these various sources together, by record linkage (see ‘Record linkage of multiple sources of data’ section), could significantly improve our knowledge of morbidity levels, patterns, and trends.

## Data sources: health status

Individual health status can be recorded by census or, better, by national health surveys either by interview or by examination. Other surveys are focused on monitoring functional or cognitive limitations and dependency in the older population.

### The census

As censuses traditionally collect data on a large variety of topics, they are ill-suited for in-depth observation. Therefore, the censuses which have in the past collected data on health (such as the UK, Belgium, Canada, Australia and New Zealand) restrict the questions on health to a very small number, mainly focused on disability. For example, the Belgian 2001 census contained seven questions, one on subjective health, three on disability due to chronic disease and three on informal care for the disabled. On a worldwide basis, an effort has been made since 2001 to collect internationally comparable disability data through the UN’s Washington Group on Disability Statistics. The latter has made recommendations for a shortlist of questions to be included in censuses or surveys in order to measure disability consistently worldwide (Mitra 2013). The shortlist includes six questions—five on functional limitations (limitations in seeing, hearing, walking or climbing steps, concentrating, and communicating) and one on self-care (limitation in showering or dressing).

Of course, only the persons capable of answering the questions can be adequately enumerated by a census.^12^ For example, individuals in private households suffering from mental illness can be excluded, though some information can be obtained from, for example, the household head acting as a proxy. Coverage of the institutionalised population in collective households (such as rest homes) is also unsatisfactory in many cases, and this population is characterised by a poorer physical and/or mental health status on average.

Due to the existence in many countries of a PIN for each individual in the census, it is now possible to link census data with register data for the same individuals. For example, one can link census information with information on death certificates in order to examine the cause of death according to the socio-economic characteristics of the deceased, or else link—for each individual—national health insurance data to those in the census. The advantages of record linkage are discussed in the ‘Record linkage of multiple sources of data’ section. The issue is no longer a technical problem but rather one of the privacy and ethics concerning the use of individual information. Of course, as more and more individual data in the developed countries can be found in national registers of various sorts,^13^ the number of topics covered by a census can be significantly reduced. Holding further censuses is even being questioned in several countries. In Belgium, for example, the linkage of various administrative registers to the national population register produces information that replaces to a large extent that obtained by a census, and the latter is presently no longer being held.

Traditional population censuses have the advantage over sample surveys, such as health interview surveys, of covering the whole population of a country at a given point in time. The results generally do not have to be bracketed by a confidence interval as no sampling is usually required, though small numbers can result from breaking down the data by area, age groups, socio-economic categories, etc. More importantly, results are available for all territorial units, even for small administrative areas or population groups (e.g. the very old). Census data are used inter alia as denominators of various incidence and prevalence rates in demography, such as age- and sex-specific death rates.

On the other hand, as traditional population censuses are expensive to hold, they are taken only once in a while (e.g. every 10 years) and cannot usually be used for the regular monitoring of data, though some countries carry on yearly updates (the USA) or use a rotation (or rolling) census (France). For a discussion of developments in census-taking see Baffour et al. (2013). In addition, despite the standardisation and harmonisation efforts, there are limits to the comparability of census data across time and space due to changing concepts and definitions, such as that of ‘household’ (Coast et al. 2016). And the absence of census sampling errors is often offset by important non-sampling errors. Compared to carefully conducted face-to-face interviews in population samples, non-response can be high and census self-reporting can be rather poor, taking into account among other factors that it is usually up to the household head to fill in the census form for all household members.

### National health surveys

National health surveys can be conducted by interview or by examination. Interview surveys rely on generic interviewers asking respondents about their experience or perception of health, while examination surveys involve the collection of objective measurements (such as weight) and of biomedical markers by professionals. Health interview surveys are based on random samples; good-quality sampling is therefore a prerequisite for the representativeness of the data. Contrary to a census, the results are sample estimates of the figures for the whole population and should be accompanied by sampling error estimations. If the focus is on specific subgroups, such as the very old, it is useful to rely on (ex ante) stratified sampling with oversampling of the subpopulations with relatively fewer individuals.

If repeated regularly over time, health surveys can be used to monitor trends in the health status and characteristics of the population, but also to study changes, for example in health inequalities, over time. For example, in England, the health survey (Health Survey for England, HSE) started in 1991 and has been conducted annually since then. Each year, there is a particular focus on a population group, disease or condition. These special topics (such as respiratory health and lung function) are repeated at appropriate intervals in order to monitor changes with time. To give another example, the US National Health and Nutrition Examination Survey (NHANES) consists both of health interviews in participants’ homes and standardised physical assessments in mobile examination centres. One can therefore evaluate, as an example, the percentage of US adults with hypertension who are unaware of their hypertension.

Even in countries, such as the Nordic ones, where various national registers are in operation, health surveys remain an invaluable tool for health assessment. Information on health behaviours, such as smoking, drinking, physical activity and quality and quantity of social relations, is not available in registers and requires interviewing the population. For example, recurrent and systematic collection of data using telephone-administered questionnaires or face-to-face ones are used in several countries to assess the quality of life and behavioural risk factors. If a PIN is available for all individuals, both types of data sources (registers and surveys) can be linked together, increasing the research base. One must consider the impact of the mode of data collection on the responses, as discussed in Thompson et al. (2013).

The main problems with surveys, even if they are well done, remain the response rate and survey representativity. On the one hand, non-respondents (e.g. the institutionalised) are probably in poorer health than those who respond. On the other hand, homeless persons or immigrants for example (also possibly in poorer health) may not be included in the sampling base. In Denmark, only 59.5% of the questionnaires were fully or partially completed. The response rate was lowest in the capital and highest in North Denmark. It was lower among men (particularly young men), older women, unmarried people and non-Danes (Christensen et al. 2012). In the 2008 health survey in Belgium, out of an initial sample of 14,549 households, only 5809 households actually participated in the interview (*Enquête de santé par interview, Belgique 2008*, ISSP, Brussels). The others could not be reached or refused to take part. Does low response bias the results of a survey? Some evidence shows it does not, at least when examining the relationship between variables in a multivariate model, though response-rate bias is found for univariate distributions (Rindfuss et al. 2015). The question remains open, however. Another issue in health interview surveys is the underreporting of events related to chronic conditions. It seems that recurring events give rise to a ‘generic memory’ for the group of events, leading to a difficulty in recalling individual incidents; in time, memories lose the details and similar events tend to become melded together (Means et al. 1989). In addition, self-assessed health reports can be somewhat unreliable and inconsistent over time.

Combined with health interview surveys, a more recent project at the EU level is the European Health Examination Survey (EHES), which aims to collect nationally representative, high-quality health data that are comparable across countries and over time. According to this programme, all EU countries should cover in their survey at least the age group 25–64 years, and extending it to people 65 years and over is recommended. People living in institutions should be included whenever feasible. According to the EHES website, core measurements, which all countries should at least include, are height, weight, waist circumference, blood pressure, total and HDL-cholesterol, fasting glucose and HbA1c (glycated haemoglobin). Once again, the problem here is a low response rate in the general population. In France, the response rate in adults was around 60% in the 2006–2007 survey, though a costly home-visit strategy was used, according to the French Institute for Public Health Surveillance (*Institut de veille sanitaire*). Unfortunately, the activities of the EHES Coordinating Centre are presently limited, due to a lack of sustainable funding. Health examination surveys are a good complement to health interview surveys. Together, they can give a comprehensive picture of the health situation in a country, though possible bias due to low response rates must be taken into account.

Concerning international comparability at the EU level, a major project has been the European Health Interview & Health Examination Surveys Database, which has maintained a record of the characteristics of major health (interview and examination) surveys in Europe. Its main objectives were among others to gather information on health survey design, questions and examination protocols: to assess and enhance the comparability of health surveys and to standardise health surveys at a European level. It is the task of the EU’s Expert Group on Health Information to ensure the consistency of data across countries in the health information field. Comparisons between countries can be affected, among others, by the fact that some surveys accept proxy responses on such items as functional limitations and the prevalence of chronic diseases, while others do not. More specific projects, at the international level, on health expectancies include Euro-REVES and the European Health and Life Expectancy Information Systems (EHLEIS) Project.

More recently, with a view of reducing costs, surveys have been held through the internet, either on spontaneous samples of volunteers or on samples of respondents recruited by phone. An example of a sophisticated approach using the capabilities of the smartphone is Stanford University’s My Heart Counts application for iPhone. A major problem is the questionable representativeness of many of these surveys. In particular, it is often not possible to extrapolate the results to a reference population, thus greatly restricting their relevance.

In a context of population ageing, some specific longitudinal surveys have been implemented for monitoring functional or cognitive limitations and dependency in the older population. For example, the Longitudinal Aging Study Amsterdam (LASA) has studied physical, emotional, cognitive and social functioning in late life, the connections between these aspects and the changes that occur in the course of time (LASA website www.lasa-vu.nl). It started in 1992 and is based on a nationally representative sample of older adults in private households aged 55 years and over.^14^ Three birth cohorts (1991–92, 2002–03, 2012–13) are followed in successive waves every 2 or 3 years. Each wave has three components: a main interview, a self-report questionnaire and a medical interview. The data collection includes measures for each of the four domains: physical, cognitive, emotional and social functioning. Biomaterial measurements, such as blood samples, are also included. For more information see Hoogendijk et al. (2016).

One can also point out the English Longitudinal Study of Ageing (ELSA), started in 2002–03, focusing on individuals over 50 living in private households, with respondents drawn from the Health Survey for England*.* Waves are held every 2 years, collecting demo-economic data and data on disability, health behaviours, cognitive functioning and mental health, with biological markers, physical variables, and performance data collected every 4 years. Another example is the Swedish National Study on Aging and Care in Kungsholmen (SNAC-K), set up in 2001. The population considered is 60+, in private households, with new cohorts aged 60 and 81 added over the years. Waves are held every 3 or 6 years according to age. Both social and medical variables are collected.

General-purpose panel surveys dealing with older adults can include questions on dependency. For example, the Generations and Gender Programme (GGP) on the consequences of demographic change, dealing with persons aged 18 to 79, collects some information on activities of daily life and on care received by the older respondents. The GGP started in the early 2000s and is conducted in 18 countries, with several waves planned. The Survey of Health, Ageing and Retirement in Europe (SHARE) started in 2004 and will be held in 28 countries in 2017. Its purpose is to collect comparative and longitudinal data (by successive waves) on health (physical, mental and cognitive status), socio-economic status and social and family networks for individuals 50+. For both the GGP and SHARE, no biological data are collected, contrary to the more specific longitudinal surveys discussed in the previous paragraphs. Moreover, these surveys are held among private households, thus excluding the institutionalised population which is more often affected by functional, sensory and cognitive impairments.

Evidently, these sources of longitudinal information are costly to run. Moreover, longitudinal surveys are affected by sample attrition over time, though samples are usually refreshed as time goes on and remaining samples can possibly be calibrated in order to reduce potential selectivity bias. Furthermore, as stressed by Hébert et al. (2012), the information derived from questionnaire-based population surveys—for the measurement of disability in an older population—is often very crude as compared to clinically based information. Comparing survey and clinical information in Québec, these authors conclude that a survey questionnaire is not a valid method for accurately estimating disability in an older population.

## Record linkage of multiple sources of data

As this paper shows, multiple sources of data on mortality, morbidity and health are now available in developed countries, and many characteristics of the individual—including socio-economic ones—are recorded. One can presently examine patterns of deaths by cause (including multiple causes of death); detect the spread of infectious diseases; monitor the incidence, prevalence and lethality of the main chronic diseases such as cancer; assess the level of disabilities and dependence—both physical and mental—of the population; measure the cost of morbidity and the consumption of medicine, etc. Recourse to these multiple sources is highly recommended, recognising that each source has its advantages but also its limits. Though the sources are to some extent complementary, the data they provide nevertheless represent a patchwork of information from which a coherent picture of mortality, morbidity and health is difficult to draw. For example, a Spanish study (Marta-Moreno et al. 2016) has compared the registration of dementia in three different sources: the pharmacy billing database, primary-care electronic health records and the hospital minimum basic dataset. The study concludes that, for this pathology at least, there is a weak concordance in the registration of dementia among the main health information systems and that all available health data sources should be taken into account in order to gain a global picture of the epidemiological and clinical reality of this health condition, which is particularly difficult to evaluate correctly.^15^

Broadly speaking, many sources of data actually refer to the same individuals, though some sources (such as surveys or sentinel networks) are restricted to population samples or to subpopulations. The same individual may, for example, be interviewed in a health survey, and later on recorded in a CVD register, with information collected by the health insurance system and hospital statistics, and end up with his/her causes of death specified on a death certificate. Being able to link these various individual records together over time is therefore a priority issue, as *record linkage* can give the event history of this individual in the areas of health, morbidity and mortality. As longitudinal samples of individuals are difficult and costly to follow up, and as retrospective information cannot be obtained once the person has died, record linkage of individual files can provide at a rather modest cost the health trajectories of individuals in the population over their lifetime. One could, for example, examine for an individual the shift from good to ill health, then to chronic disease, disability and finally death, possibly taking into account various socio-economic characteristics of the individual (such as education and employment) and their change over time. Of course, record linkage does not yield a continuous picture of one’s health situation but rather a series of shots of one’s life. If the cuts between the shots are short, the main features of the life course can nevertheless be observed.

Record linkage should ideally be performed on the basis of a personal identification number (PIN) or another individual identifier common to all data sources. If these are unavailable, the linkage can sometimes be conducted on the basis of common individual characteristics (see for example Masuy-Stroobant et al. 1977; Deboosere and Gadeyne 1999), using probabilistic matching. Linking on individual characteristics may however lead to a significant number of non-linked cases. If the census is involved, this procedure should refer to a time period close to the census. The procedure is difficult to apply for linking multiple registers together.^16^

Record linkage is of course much easier to perform if the same PIN is used in the various data sources (see, e.g. Jasilionis et al. 2007; Elo et al. 2014). With a PIN, one can link, for example, death by cause, from the vital registration system, to the socio-economic characteristics of the deceased, obtained from the censuses. In addition to individual-level matching, address-level matching has also been used, such as the Secure Anonymised Information Linkage (SAIL) system in Wales. To give another example, one can point out the additional use of both address code and business register code in Denmark for combining information on persons, dwellings and employment from different administrative sources.

One can also carry out a linkage between censuses and surveys, between multiple registers or between surveys and registers, such as do the Nordic countries, the UK, Canada and Australia. For example, a Danish study on sex-specific selection and information bias in surveys—possibly explaining the health-survival paradox, i.e. the fact that men report better health than females and yet women outlive men—has been based on the linkage of three population-based surveys with health registers: the Study of Middle-Aged Danish Twins (MADT), the Longitudinal Study of Aging Danish Twins (LSADT), the Danish 1905 Cohort Study, on the one hand, with various registers^17^ within Statistics Denmark, on the other (Oksuzyan et al. 2009).

Some caveats should however be added. First, individuals without a PIN, such as asylum seekers, will not be included in the linkage. Secondly, persons who have emigrated are lost to follow-up. Thirdly, optimum record linkage requires good collaboration among providers of data. Finally, there are important privacy issues at play.^18^ Several methods can be used to ensure privacy protection, including multistage and role-specific encryption of pseudonymised identifiers, restricted access to variables and small-number disclosure control (see, e.g. Lyons et al. 2014). An emerging and important issue is the threat of cyberattacks on highly connected computer systems, for political or criminal reasons. A ransomware cyberattack in May 2017 forced some UK hospitals to cancel operations and outpatient appointments.

## A Big Data approach

Big Data refers to very large and complex datasets generated by different means, comprising structured and unstructured data that can be examined by various methods of exploratory data analysis. Big Data can refer, among others, to the numerous electronic morbidity and health records now available and examined in this paper. They can also be drawn from the internet (e.g. from social media sites) or obtained from electronic devices such as video cameras, smart phones, etc. Even when individual matching of records is not possible, a Big Data approach is helpful in revealing changing mortality and morbidity patterns in time and place—just as it can be used in other fields for the early detection of defects—in view of possibly taking preventive measures rather than more costly curative ones. In the field of morbidity, Big Data could lead to a better identification of the risk factors of a disease, improve diagnostics, help in choosing the more relevant drugs and in monitoring the efficacy of treatments, etc.

For example, Ramos-Casals et al. (2015) used the Google search engine to collect and merge large series (> 1000 patients) of systemic autoimmune diseases (SAD) reported in the PubMed library. The study, covering nearly 400,000 patients with SAD, showed different patterns by gender, age and geographical distribution by type of SAD. In the field of infectious diseases, the use of Big Data at the level of the individual can lead to early detection of new cases of a disease or of the emergence of a new disease, an application of query-based outlier detection in heterogeneous information networks. For example, Germany has implemented an automated outbreak detection system that monitors the routinely collected surveillance data for communicable diseases. The system detects unusually high case counts and is based on state-of-the-art statistical procedures for data mining (Salmon et al. 2016).

More generally, Big Data can reveal patterns in the data and possible anomalies*.* Another advantage of Big Data lies in the size of the dataset—the larger the size, the more power available for performing a test. In other words, a larger sample size reduces the variance of the estimate. Large samples can also yield better coverage of the population of reference and can detect subsets of data requiring more specific modelling. Methods of handling data with varying degrees of precision are improving, as are those for efficient query evaluation and probabilistic database research. For example, Marucci-Wellman et al. (2015) used Naïve Bayes algorithms for semi-automated coding of short injury narratives from large administrative databases. Auto-measurement of health indicators by the individual him/herself can be taken following a standardised protocol and included in an electronic database, with feedback to the individual validated by scientific studies. For example, new technology such as the smartphone^19^ can be used to monitor various health characteristics and to automatically transmit this information to a central database, where medical feedback can be provided. Social media data, such as social networking sites, have been used, for example, for research on perceived risk factors of type 1 diabetes. Moreover, cloud computing can provide the huge computing and storage resources needed for Big Data.

Of course, when several types of information are examined in the same source of data, the application of Big Data research to health care is even more promising if the same individual identifier (PIN) is attached to the data. To give an example based on Murdoch and Detsky (2013), electronic health records contain not only quantitative data, such as laboratory values, but also qualitative data, such as text-based documents, and transactional data, such as medication delivery. Analysing these structured and unstructured data at the individual level can provide fruitful knowledge, for example on postoperative complications or interaction among drugs, that can guide future clinical decisions.

The recourse to personalised information from multiple sources of data can bring even greater rewards. Individual data are now being increasingly collected by a variety of means on a range of subjects, such as genomics, medical imaging, clinical diagnoses and physiological sensing. In all these individual fields, the mining of the Big Data collected can provide results conducive to more accurate and efficient healthcare; a series of examples of such studies is given in Herland et al. (2014). The most promising perspective, however, is to explore the data from these various sources together; integrating individual data gathered at multiple levels in view of improving diagnostics, prognostics and therapies and of producing new indications for personalised treatments. The field of translational bioinformatics is currently being developed to accomplish this (see among others Andreu-Perez et al. 2015). According to the American Medical Informatics Association, translational bioinformatics is the development of storage, analytic and interpretive methods to optimise the transformation of increasingly voluminous genomic and biomedical data into proactive, predictive, preventive and participatory health (https://www.amia.org).

In demography, morbidity and mortality microdata are collected at the individual level by a variety of sources discussed previously in this paper. These sources include medical records held by GPs, specialists and other practitioners in the health field, hospital records, pharmaceutical drug consumption, disease register data, death certificates and autopsy examination. If this diverse information could be linked together, it could improve, for instance, multiple cause-of-death reporting. To give another example, mobile phone and GPS data have been used to provide information on population displacements in cases of crises, such as hurricanes (as in Haiti in 2016) or earthquakes (as in Nepal in 2015), as does the non-profit Flowminder Foundation. As pointed out by Steven Ruggles (2014, p. 293), other promising topics of investigation using ‘Big’ microdata in demography include residential segregation, migration and migrant settlement patterns, rural depopulation and agricultural consolidation, the identification of concentrated poverty, possible causes and levels of change in ecosystems as a function of human-environment interactions, comparative cross-national policy analysis and multilevel analysis of the impact of community characteristics on individual behaviour.

Issues remain nevertheless in a Big Data approach (Kuhn and Johnson 2014), such as the protection of individual privacy and avoiding disclosure of confidential information. In the EU, Directive 2016/680, which will enter into force in May 2018, aims at protecting individuals with regard to the processing of personal data. In particular, the Directive states that personal data concerning health should include all data pertaining to the health status of a subject which reveal information relating to the past, current or future physical or mental health status of the subject (Article 24). Privacy issues are especially relevant when the database is privately owned, as in the case of some large genetic bio/databanks (e.g. 23andMe). Moreover, analyses are complicated by the fact that data are not stored in a centralised location and by the sheer amount of data to be stored and processed, at great financial cost. For example, the Cancer Genome Atlas (TCGA) dataset is over a petabyte in size and consists of more than 575,000 files. Downloading the data using a 10-Gbit-per-second connection would take over 3 weeks (Grossman et al. 2016). It has even been argued that ‘forgetting’ or shedding information should be part of today’s data management, particularly for techniques requiring fast query answers (Heinis and Ailamaki 2015).

A related issue is sparseness. To give but one example, electronic health records contain only some details for each patient while many fields are null; this leads to wasting a huge amount of memory in data mining. Standardised methods for storing the data in a common format are therefore required (Batra and Sachdeva 2016). Turning Big Data into knowledge remains, at present, a critical challenge. Exploratory analysis, such as data mining; information visualisation and machine-learning of big databases solely yields possibly spurious associations among variables and not causal links. While an exploratory-data approach is never a substitute for sound causal modelling, it can nevertheless usefully inform it, especially when background knowledge on the topic of interest is scant. A good (i.e. bad!) example of erroneous causal inference is the well-known Google Flu Trends study, matching search terms and flu propensity; the structurally unrelated matches were much too numerous (Lazer et al. 2014). And Big Data does not guarantee the absence of systematic bias if the primary sources themselves are biased, thus the need for a thorough evaluation of the quality of these sources for scientific purposes. In particular, one must know by whom and for what purpose the databases have been made.

## Conclusions

To conclude, numerous sources of data on mortality, morbidity and health are available in advanced societies.^20^ It is important that these data be archived and safeguarded, notably for privacy reasons, against intrusion. In particular, an argument for protection—as pointed out earlier—is that interconnected computer systems, such as those being developed in the health field, are highly at risk of cyberattacks for political or criminal motives.

While each source considered separately can already yield useful, though partial, results, record linkage among data sources with a common identifier can significantly improve the overall picture of levels and trends in mortality and health, serving as a basis for better projections/scenarios in these fields. Record linkage can also lead to the exploratory or confirmatory analysis of possible causal associations between the outcomes in question and various micro-macro characteristics of the individual and his/her environment. Big (structured and unstructured) Data can reveal changing mortality and morbidity patterns in time and place and can lead to health policies that take preventive measures rather than more costly curative ones. Moreover, physicians can extract useful clinical information from Big Data to obtain, for instance, more detailed medical histories and improve personalised treatment plans, leading to lower morbidity, better patient care and reduced costs. In demography, as pointed out in the ‘A Big Data approach’ section, numerous topics of research could possibly benefit from a ‘Big’ microdata approach, considering the increasing abundance of digital individual-level data. However, this area remains to be explored.

Information exchange requires enhancing interoperability among the health information systems that are managed by different organisations in each country. Moreover, ensuring comparability of data, across countries and over time, remains an issue despite UN, WHO and EU efforts to address this. Ethical concerns are also not to be overlooked.

## Footnotes

1 See Supplement 3 of the *American Journal of Preventive Medicine* (2016), volume 51, issue 5.

2 It is estimated, for example, that antibiotic-resistant infections are associated with 23,000 deaths in the USA each year (Jaffe 2014). Drug-resistant bacteria are moreover one of the causes of sepsis.

3 Other sources of data collection on infectious diseases or of environmental surveillance of viruses (e.g. poliovirus), such as sewage surveillance employing manual or automatic sampling, will not be discussed here.

4 The weekly online journal *Eurosurveillance*, published by ECDC, is an excellent source of information on surveillance and outbreaks of infectious diseases in the EU.

5 A relevant example is the weekly maps established by the ECDC of reported autochthonous human cases of West Nile fever in Europe and the Mediterranean basin.

6 Such as whole-genome sequencing of microbes.

7 See ‘sentinel surveillance’ at www.who.int.

8
http://www.ktl.fi/publications/monica/manual/index.htm

9 Around 400 population-based operational cancer registers, with follow-up of registered cancer patients, have been identified worldwide (as of 2009) (Allemani et al. 2015).

10 For example, in the USA, the percentage of hospital emergency departments with an EHR system has increased from 46% in 2006 to 84% in 2011 (Jamoom and Hing 2015).

11 We do not consider here the private dependency insurance schemes which are increasingly available.

12 The same is true for sample surveys, discussed in the ‘National health surveys’ section.

13 Including the national (or central) population register in several countries; see Poulain and Herm (2013).

14 Nationally representative sample obtained from the registries of 11 municipalities across three culturally distinct regions in the Netherlands.

15 In the case of mental diseases, the American Psychiatric Association edits a *Diagnostic and Statistical Manual of Mental Disorders*, presently in its 5th revision.

16 A thorough evaluation of the quality of the Belgian 2011 ‘census’, based on a linkage of various registers, remains to be done.

17 The Danish Demographic Database, the National Patient Register and the Prescription Medicine Register.

18 As an example of the legal issues involved see for instance Lacour (2016).

19 For example, the AliveCor Heart Monitor consists of a smartphone app plus a phone case with special sensors that can detect possible atrial fibrillation.

20 For example, there are around 260 public databases on health in France, according to INSERM (see *Big data en santé* on the INSERM website, at www.inserm.fr).
